# Bayesian Generative Models for Knowledge Transfer in MRI Semantic Segmentation Problems

**DOI:** 10.3389/fnins.2019.00844

**Published:** 2019-08-21

**Authors:** Anna Kuzina, Evgenii Egorov, Evgeny Burnaev

**Affiliations:** Center for Computational and Data-Intensive Science and Engineering, Skolkovo Institute of Science and Technology, Moscow, Russia

**Keywords:** brain tumor segmentation, brain lesion segmentation, transfer learning, Bayesian neural networks, variational autoencoder, 3D CNN

## Abstract

Automatic segmentation methods based on deep learning have recently demonstrated state-of-the-art performance, outperforming the ordinary methods. Nevertheless, these methods are inapplicable for small datasets, which are very common in medical problems. To this end, we propose a knowledge transfer method between diseases via the Generative Bayesian Prior network. Our approach is compared to a pre-train approach and random initialization and obtains the best results in terms of Dice Similarity Coefficient metric for the small subsets of the Brain Tumor Segmentation 2018 database (BRATS2018).

## 1. Introduction

Magnetic resonance imaging (MRI) is a medical imaging technique used in radiology to form pictures of the anatomy of some part of the human body. It is used as a diagnostic tool for various types of cancer, diseases of the central nervous system, such as multiple sclerosis or epilepsy (Hammers et al., 2007; Sharaev et al., 2018a,b), depression (Sheline, 2000; Ivanov et al., 2018) and in plenty other cases (Ronneberger et al., 2015; Çiçek et al., 2016). Recent advances in computer vision revealed a high potential for application of neural networks in the medical problems: classification of MRI or CT for disease diagnosis, automatic detection and segmentation of different pathologies (Gong et al., 2007; Davatzikos et al., 2008; Pominova et al., 2018). Even though it is unlikely that these models will be used as a diagnostic tool without any human intervention in the nearest future, they could be beneficial serving as decision support systems.

Semantic segmentation of MRI scans is an essential but highly challenging task. Accurate segmentation can simplify and speed up the work of radiologist, reduce the risk of mistakes by automatic detection of tumors (Kohl et al., 2017), multiple sclerosis plaques (Rey et al., 2002), hemorrhages (Davuluri et al., 2012; Guerrero et al., 2018) or other disease manifestations (Wachinger et al., 2018). It is also applicable for analysis and quantification of some illnesses. For example, currently, the exact volume of affected brain areas of patients with multiple sclerosis is not calculated due to the extreme difficulty of this task. Instead, a very rough approximation is used while exact information about affected volumes in practice may be highly useful for understanding the progression of the disease.

State-of-the-art methods for semantic segmentation imply the use of deep neural networks, which usually have millions of tuning parameters, hence demanding a large amount of labeled training samples to avoid overfitting. At the same time, manual labeling of the MRI with tumors or other manifestation of the disease, is time consuming and expensive. Consequently, in most cases only tiny datasets are available for training. As a result, methods which need less labeled examples for training are of great significance. To this end, we can exploit knowledge from existing labeled datasets.

Medical imaging dataset has several crucial peculiarities, which one should take into account while solving semantic segmentation problem with the small training dataset. We can group them into image preprocessing, prediction postprocessing, selection of network architecture and specificity of the transfer learning from data with a different disease. Preprocessing includes image alignment, skull-stripping, normalization of the images within a given dataset (Litjens et al., 2017). A variety of MRI protocols are available with or without the use of contrast agents. These protocols allow the setting up of different contrasts among the various tissues within the same organ system. Thus, the quality of the segmentation heavily depends on this feature of the dataset.

Depending on the dataset, different postprocessing of the obtained prediction may be required. For example, it is a common problem, that the full 3D scan does not fit into memory, and one has to use patches to obtain predictions. Predictions for overlapping patches are further combined by giving a higher weight to the pixels in the center since they are known to produce better predictions. Moreover, for some problems, it is known that predicted mask could not contain more that one connected component, e.g., when a separate organ or it's part is being segmented. In this case, postprocessing could also remove all the extra prediction, which may drastically boost the performance.

Furthermore, the choice of network architecture is a crucial step. Semantic segmentation problem is usually solved in computer vision by fully convolutional networks with architectures similar to U-Net (Ronneberger et al., 2015). U-Net with 3D convolutions also known as V-Net (Milletari et al., 2016) is extensively applied to various types of medical images (Ronneberger et al., 2015; Milletari et al., 2016; Deniz et al., 2018; Guerrero et al., 2018; Livne et al., 2019). The state-of-the-art approaches consider additional regularization with training multitarget networks and also the ensembling of the models (Myronenko, 2018) or cascade models by stacking several V-Nets (Isensee et al., 2018).

Finally, there is a common practice to apply transfer learning techniques, when the size of the target training dataset is not sufficient. There exist several large publicly available datasets with labeled segmentation, which may be used to transfer knowledge to smaller ones. Nevertheless, these images may be pretty different in terms of diseases, modality, protocols and preprocessing methods, which leads to extra difficulties. In this work, we address the problem of knowledge transfer between medical datasets when source dataset potentially contains relevant information for the given problem (e.g., it depicts scans of the same organ), but still comes from the different domain, complicating the work of the conventional transfer learning techniques.

### 1.1. Transfer Learning Approach

Transfer learning is a set of techniques from machine learning, used to store knowledge from one problem or dataset and apply it to another but similar problem (Pan and Yang, 2010). In deep learning, it is usually performed by network initialization with weights trained on source dataset and fine-tuning on a target dataset. If the size of the target dataset is too small, some parameters of the network may be frozen to avoid overfitting. This approach can be beneficial for the segmentation of medical images (Havaei et al., 2016), but the degree to which it will be useful highly depends on the source and target datasets similarity. Van Opbroek et al. (2015) applied transfer learning to support vector machine classifier in the setting, where the source and target datasets only differ in scanners and acquisition protocols. The authors showed that with a small target dataset transfer learning considerably outperforms common supervised learning approach. Ghafoorian et al. (2017) were also using very similar datasets for transfer learning in white matter hyperintensities segmentation problem and obtained higher dice similarity coefficient when the model was trained on the target and fine-tuned on the source domain. The authors of both papers assumed that source dataset is almost the same as a target one with only small differences, such as scanner type or voxel size to be present.

Margeta et al. (2017) used fine-tuning to solve classification task on MRI scans. A convolutional neural network was pre-trained on a dataset with natural images, which is somewhat irrelevant for their problem and therefore requires fine-tuning of the whole model with a relatively big dataset of 215 MRI scans. Zhou et al. (2017) proposed using continuous fine-tuning when training dataset is steadily expanded with images, labeled by the current version of the model. The authors suggested starting from the pre-trained network and choosing the most confident predictions of the model to include them into the training set. The main restriction of this approach is the fact that the method requires unlabeled data from the same domain. Moreover, the authors suggested working only with patches of images to assess the confidence of the algorithm, which might be less practical for tasks different from classification, such as detection or segmentation. Han et al. (2018) exploited network pre-trained on a large number of X-ray computed tomography (CT) to restore high-resolution MRI from under-sampled k-space data with few training MR observations available.

Christodoulidis et al. (2017) showed improvement in lung tissue pattern classification accuracy when fine-tuning the model trained on six open-source texture databases separately and taking an ensemble of all these models. The authors determined that transfer learning from a single dataset does not provide a stable increase in accuracy and sometimes even performs worse than random initialization. Li et al. (2018) proposed a novel approach, which helps to transfer knowledge from healthy subjects to new disease classification problem. They showed improvement in accuracy, sensitivity, specificity over deep neural network trained from scratch. But the authors only use fully connected layers, working with features exctracted from the functional MRI, rather that with raw images.

Another branch of work suggests dealing with smaller sample size, using mixed supervision models (Mlynarski et al., 2018; Shah et al., 2018). These papers highlight that for medical image segmentation we cannot rely on transfer learning of parameters from networks (pre-)trained for analysis of natural images. Hence, the authors proposed to simultaneously use high-quality expensive labeling with lower-quality but cheap labels for training (mixed supervision). Despite of the interesting results, the developed method considers the case of partially available expensive labels from one dataset. However, we consider the case of different datasets with different diseases.

Transfer learning may be also considered as a special case of the domain adaptation problem (Wilson and Cook, 2018), when one aims to take model trained on one domain (referred to as source) and adapt it to perform just as well on a new target domain.

Finally, Elsayed et al. (2018) suggest novel approach, adversarial reprogramming of the neural networks. The paper considers an additive perturbation to the network input to apply the adversarial reprogramming. The authors demonstrated adversarial reprogramming on classification tasks in the 2D image domain (MNIST classification, and CIFAR-10 classification). To apply the approach one should define a hard-coded mapping function from source labels to the adversarial task labels. Therefore we can not apply this approach to the segmentation tasks. Moreover, the method is applicable only for datasets with images of smaller spatial size than that of the source dataset. Hence, it is an interesting research problem to adapt the proposed technique for segmentation tasks of 3D MR images, however, it is out of the scope of our paper.

In this paper, we propose a method for knowledge transfer between diverse neuroimaging datasets. Conceptually, our approach consists of the following steps: we solve the semantic segmentation problem for a small labeled training dataset. Provided a larger dataset, referred to as the source, which may differ from the target dataset drastically in terms of the modality, resolution or other properties. Proposed method outperform straightforward fine-tuning on studied semantic segmentation problem.

When dealing with a small dataset along with the multidimensional model, there exists a high risk of overfitting. Experiments show that filters of different segmentation networks often exhibit similar structure, which could be exploited for regularization purposes. Probabilistic formulation of the model allows us to apply these restrictions on the weights formally using the method described below.

At the first stage, the source dataset is used to train a segmentation network. Following the assumption that kernels from this model have a useful structure for the target segmentation problem, generative model—Variational Autoencoder (VAE) (Kingma and Welling, 2014) is trained on the weights from the source network which tries to approximate the distribution of the kernels. Finally, to solve the target problem, we fit the segmentation network with the same architecture as in the first point but with the generative model used as a prior distribution over the weights.

The rest of the paper is organized as follows: in sections 2.1–2.3 we discuss U-Net architecture (Ronneberger et al., 2015), which was used for semantic segmentation, describe deep Bayesian approach for training neural networks with prior distribution over parameters and, finally, explain how we can learn prior distribution from data and apply it to variational inference to perform knowledge transfer. Section 2.4 is devoted to the medical datasets, that were used for the experiments, in sections 2.5–2.7 more practical details, such as metrics, loss functions and experimental setup are presented. Section 3 discusses the results of the experiments, where we compare the proposed approach with random initialization and pre-trained weight initialization. Finally, in section 4 we discuss the key findings of the study, potential drawbacks and outline for the future work.

## 2. Materials and Methods

In this part, we shall discuss U-Net architecture, which serves as a foundation for all the experiments in this work. Then we discuss the approximate Bayesian approach, stochastic variational inference (Hoffman et al., 2013) in deep neural networks and the importance of prior distribution selection. This part is crucial for the understanding of Deep Weight Prior (DWP) (Atanov et al., 2018), which allows us to transfer knowledge among datasets. The idea of DWP lies in the fact that we learn the prior distribution of convolutional filters in the form of a generative model, instead of using parametric distribution. Since we get kernels from the network trained on source dataset to learn the prior and further exploit it for variational inference on the target dataset, this approach can be considered as a transfer learning technique. Finally, we proceed to the description of the practical part, including datasets, validation methods, loss function and complete experiment setup, which evaluates the performance of the proposed approach.

### 2.1. 3D U-Net

**Figure 1:**
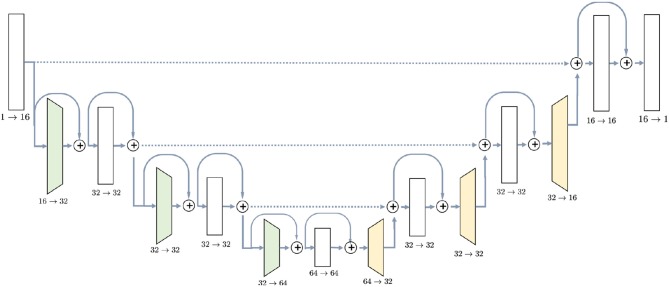
U-Net architecture used in the experiments contains ResNet-like blocks both in Encoder and Decoder parts with skip-connections.

U-Net (Ronneberger et al., 2015) was chosen due to its popularity and experimentally proven efficiency for MRI semantic segmentation tasks (Milletari et al., 2016; Deniz et al., 2018; Guerrero et al., 2018; Livne et al., 2019). The detailed architecture of the network is shown in Figure 1. It consists of downsampling blocks, colored in green, upsampling bocks (yellow) and simple blocks which do not change spatial resolution of the image. The chosen architecture has 726,480 parameters, estimated from a training set of 20 or 10 images. Since U-Net is a fully convolutional network, the number of parameters does not depend on the input size. Regardless of the initial resolution, each input is compressed by the factor of 8 in the encoder part of the network and upsampled back to the initial size in the decoder. For instance, BRATS18 (Menze et al., 2015) which is initially cropped to [152, 184, 144] pixels, gets compressed to the [19, 23, 18] in the middle of the network and then decoded back to the initial size.

The first part, encoder, takes an image as the input and gradually decreases its resolutions, using strided convolutions, and simultaneously increases the number of channels. Each block in the encoder, except for the initial one, consists of two convolutions with kernel size 3 × 3 × 3, preceded by instance normalization and ReLU activation layer. Downsampling blocks differ only in the sense that the first convolution has stride 2, instead of 1. Blocks have ResNet-like structure (Guerrero et al., 2018) when the input is added to the output of the block.

Decoder, on the other side, steadily increases the spatial resolution of the image to return it to the initial size. Upsampling block does not have a residual connection, and it consists of one 3D convolution with kernel 3 preceded by instance normalization and ReLU activation and is followed by a trilinear upsampling with factor 2. Simple blocks are identical to the encoder part, except that they take as input not only the output of the previous layer but also an output of the encoder block with the same resolution. This feature of the U-Net model, known as skip-connection, allows the model to keep more details in the reconstruction.

### 2.2. Deep Bayesian Models

In this part, we present deep Bayesian Inference and apply it to the U-Net model. Consider a U-Net model with *L* convolutional layers. Denote by *w*^(*i*)^, *i* = 1, …, *L* kernels for the *i*th convolutional layer and *w* = (*w*^(1)^, …, *w*^(*L*)^) vector of all the model parameters. If kernel filters at a layer *i* are of size 3 × 3 × 3, with $C_{inp}^{\left(i\right)}$ input channels and $C_{out}^{\left(i\right)}$ output channels, then the weight matrix has dimensions of $C_{inp}^{\left(i\right)}\times C_{out}^{\left(i\right)}\times 3\times 3\times 3$.

In the Bayesian approach, one combines prior distribution *p*(*w*) on the parameters *w* of the model with the information from observed training dataset $D={\left\{\left(x_{i},y_{i}\right)\right\}}_{i=1}^{N}$ in the form of likelihood p(D|w) by posterior distribution p(w|D), computed with Bayes formula:

$$\begin{array}{l} p\left(w|D\right)=\frac{p\left(D|w\right)p\left(w\right)}{p\left(D\right)}. \end{array}$$

For most cases, posterior distribution cannot be computed in closed form, since denominator of the above formula is not tractable. A common way to deal with this problem is to apply variational inference (Jordan et al., 1999) when posterior is approximated by parametric distribution *q*_θ_(*w*) which minimizes Kullback-Leibler divergence between the true posterior p(w|D) and its variational approximation *q*_θ_(*w*). More specifically, we are not interested in a point estimate of the model's weights *w*. Instead we are going to receive its distribution which is parametrized by θ.

Moreover, we assume that both variational approximation *q*_θ_(*w*) and prior distribution *p*(*w*) are factorized over layers, input and output channels:

$$\begin{array}{ll} q_{\theta }\left(w\right) & =\overset{L}{\underset{i=1}{\prod }}\overset{C_{inp}^{\left(i\right)}}{\underset{p=1}{\prod }}\overset{C_{out}^{\left(i\right)}}{\underset{k=1}{\prod }}q_{\theta_{ipk}}\left(w_{p,k}^{\left(i\right)}\right),\\ p\left(w\right) & =\overset{L}{\underset{i=1}{\prod }}\overset{C_{inp}^{\left(i\right)}}{\underset{p=1}{\prod }}\overset{C_{out}^{\left(i\right)}}{\underset{k=1}{\prod }}p\left(w_{p,k}^{\left(i\right)}\right). \end{array}$$

Given all the assumptions above, the task burns down to the maximization of evidence lower bound (ELBO) (Hoffman et al., 2013) with respect to parameters of variational posterior distribution θ:

(1) $$\begin{array}{l} \underset{\theta }{\max}\;L\left(\theta \right)\\ \approx \underset{\theta }{\max}L_{D^{*}}-\sum_{i,p,k}\underset{\text{KL-divergence between }q_{\theta_{ipk}}(w_{p,k}^{\left(i\right)})\text{ and }p(w_{p,k}^{\left(i\right)})}{\underset{︸}{\int q_{\theta_{ipk}}(w_{p,k}^{\left(i\right)})\log\left(\frac{q_{\theta_{ipk}}(w_{p,k}^{\left(i\right)})}{p(w_{p,k}^{\left(i\right)})}\right)}}. \end{array}$$

Detailed derivation of the above expression is presented in Appendix A. The first part of the formula is a data term $L_{D^{*}}$, also referred to as a reconstruction error. It is in charge of prediction quality, forcing the model to fit the data. Second term—Kullback–Leibler divergence between variational distribution and prior over parameters of the model requires posterior distribution to be as close as possible to the prior, serving among other things as a regularization.

In the Bayesian framework, prior distribution is used to incorporate some knowledge or specific property, such as sparsity (Neklyudov et al., 2017) into parameters of the model. In the context of the current work, we consider prior distribution as a method for knowledge transfer. During our experiments with MRI semantic segmentation, we have noticed that kernels from different segmentation networks share a similar structure, when appropriately trained, in contrast to noisy kernels from models trained on small datasets. Therefore, prior distribution, which restricts kernels to be more structured, presumably should improve segmentation quality on modest training sets. We propose to apply Deep Weight Prior, discussed in the next part, to enforce precisely this property.

### 2.3. Deep Weight Prior

Deep Weight Prior (Atanov et al., 2018) is an expressive prior distribution, which helps to incorporate information about the structure of previously learned convolutional filters during training of a new model. Prior is learned in the form of a generative model—Variational Autoencoder (Kingma and Welling, 2014). It allows us to learn expressive distribution over the kernels, but we do not have direct access to its density and are only able to obtain samples.

Priors, whose probability density function (pdf) *p*(*w*) is not accessible directly are called implicit in contrast to explicit priors, where pdf is available. To work with implicit priors we introduce some latent variables, assuming that conditional distribution with respect to them comes from some parametric family e.g., Gaussian distribution. We will use this method to work with Deep Weight Prior.

More precisely, we will consider implicit prior distribution in the form of Variational Autoencoder (VAE) (Kingma and Welling, 2014) with encoder $r_{\psi^{\left(i\right)}}\left(x|w\right)$ and decoder $p_{\phi^{\left(i\right)}}\left(w|z\right)$, modeled by neural networks. Finally, given the prior over latent space *p*(*z*), we arrive at the prior distribution for the kernels from the layer *i*:

$$\begin{array}{l} p^{\left(i\right)}\left(w\right)=\int p_{\phi^{\left(i\right)}}\left(w|z\right)p\left(z\right)dz. \end{array}$$

The main advantage of this prior is that it is non-restrictive, learnable from data and provides a fast sampling opportunity. Unfortunately, with implicit prior, it is not possible to compute Kullback–Leibler divergence from the ELBO objective (Equation 1). To this end, we follow the work of Atanov et al. (2018) which replace KL-divergence by its upper bound.

$$\begin{array}{r} \text{KL}\left(q_{\theta }\left(w\right)||p\left(w\right)\right)\leq {\text{KL}}^{\text{approx}},\\ {\text{KL}}_{i,p,k}^{\text{approx}}=-ℍ(q_{\theta_{ipk}}(w_{p,k}^{\left(i\right)}))+E_{q_{\theta_{ipk}}(w_{p,k}^{\left(i\right)})}[\text{KL}(r_{\psi^{\left(i\right)}}(z|w_{p,k}^{\left(i\right)})||p^{\left(i\right)}(w))-E_{r_{\psi^{\left(i\right)}}(z|w_{p,k}^{\left(i\right)})}\log p_{\phi^{\left(i\right)}}(w_{p,k}^{\left(i\right)}|z))], \end{array}$$

where ℍ(·) is an entropy of a corresponding distribution.

If $r_{\psi^{\left(i\right)}}\left(x|w\right)$, $p_{\phi^{\left(i\right)}}\left(w|z\right)$ and *q*_θ_(*w*) are explicit distributions, we can use approximate lower bound (equation 2), for which we will be able to compute stochastic gradients with reparametrization trick to perform stochastic variational inference. We maximize approximate ELBO with respect to the parameters of the variational posterior distribution θ and DWP encoder parameters ψ.

(2) $$\;\underset{\theta ,\psi }{\max}L{\left(\theta \right)}^{\text{approx}}=\underset{\theta ,\psi }{\max\;}L_{D}-{\text{KL}}^{\text{approx}}$$

**Figure 2:**
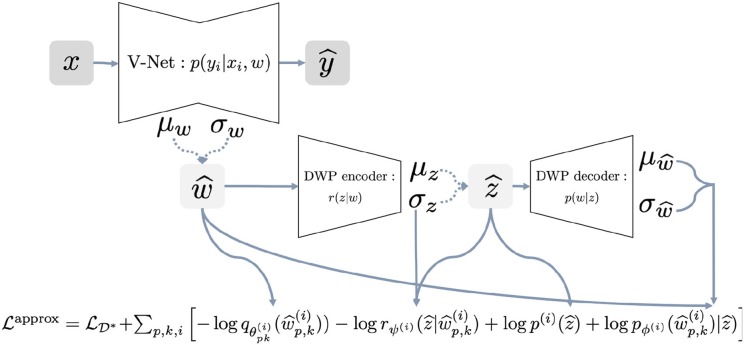
Full scheme of the training procedure with Deep Weight Prior.

The Algorithm 1 provides a pseudocode for the proposed algorithm. The algorithm requires as input the trained variational autoencoder on the reference dataset. We discuss particular details of training in section 2.7. Details on how different parts of the loss function are calculated, are presented in the Figure 2 for better understanding. We begin with sampling weights with reparametrization from variational distribution, which is fully factorized Gaussian $\hat{w}\;\sim \;q_{\theta }\left(w\right)$. These samples are used to compute log-density of the variational posterior and parameters of the distribution $r_{\psi^{\left(i\right)}}\left(z|\hat{w}\right)$. Distribution $r_{\psi^{\left(i\right)}}\left(z|\hat{w}\right)$ is used to sample with reparametrization latent variable $\hat{z}\;\sim \;r_{\psi^{\left(i\right)}}\left(z|\hat{w}\right)$ to further pass it to the decoder and obtain parameters of the distribution $\log p_{\phi }\left(\hat{w}|\hat{z}\right)$. At this point, we have all the components of the objective to calculate stochastic gradient and update parameters θ of the U-Net and ψ of the DWP encoder.

**Algorithm 1:** Algorithm for training model with Deep Weight Prior.

**Input:** Dataset $D:{\left\{\left(x_{i},y_{i}\right)\right\}}_{i=1}^{N}$
**Input:** Variational approximation of the posterior distribution *q*_θ_(*w*) **Input:** DWP with encoder *r*_ψ_(*z*|*w*) and decoder *p*_ϕ_(*w*)
while not converged **do**
Sample minibatch $D^{*}:{\left\{\left(x_{i},y_{i}\right)\right\}}_{i=1}^{M}$
**for** Layer *i* ∈ {1, …*L*}, input channel *p* ∈ {1, …*C*_*inp*_} and output channel *k* ∈ {1, …*C*_*out*_} **do**
Sample weights with reparametrization: ${\hat{w}}_{pk}^{\left(i\right)}\sim$ $q_{\theta_{ipk}}\left(w_{pk}^{\left(i\right)}\right)$
Sample latent variables with reparametrization: ${\hat{z}}_{pk}^{\left(i\right)}\sim$ $r_{\psi^{\left(i\right)}}\left(z|{\hat{w}}_{pk}^{\left(i\right)}\right)$
Compute stochastic gradients of the objective:
$\begin{array}{l} L^{\text{approx}}=L_{M}+\sum_{p,k,i}[-\log q_{\theta_{ipk}}({\hat{w}}_{p,k}^{\left(i\right)}))-\log r_{\psi^{\left(i\right)}}(\hat{z}|{\hat{w}}_{p,k}^{\left(i\right)})\\ +\log p(\hat{z})+\log p_{\phi^{\left(i\right)}}({\hat{w}}_{p,k}^{\left(i\right)}|\hat{z})] \end{array}$
Update parameters $\theta =\theta +\alpha {\;▽}_{\theta }{\;L}^{\text{approx}}$ and ψ = $\psi +\beta {\;▽}_{\psi }{\;L}^{\text{approx}}$
**Output:** *q*_θ_(*w*) — posterior distribution of the model parameters

### 2.4. Datasets

To emphasize the ability of the proposed approach to generalizing, two public available datasets were chosen with different diseases on the challenging task of the brain segmentation.

**Figure 3:**
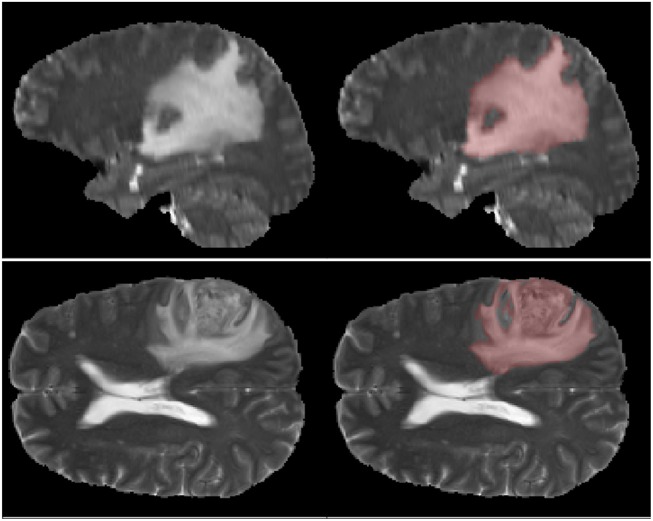
Example of MRI slices and ground truth segmentation from BRATS18 dataset.

**Figure 4:**
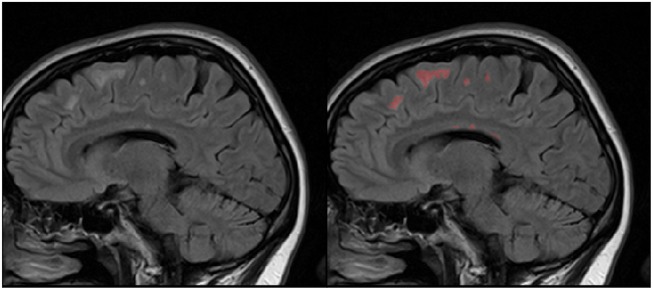
Example of MRI slices and ground truth segmentation from MS dataset.

First one comes from the annual competition on brain tumor segmentation, BRATS18 (Menze et al., 2015; Bakas et al., 2017). It contains pre-operative MRI scans of 275 patients with glioblastoma (GBM/HGG) and lower grade glioma (LGG). Each volume has resolution 240 × 240 × 155 pixels, acquired with different protocols and scanners in 19 institutions. All the images were co-registered, reshaped to the same resolution and skull-stripped. Ground truth labels were manually created by expert neuroradiologists for all the scans. The analysis was performed on T2-weighted volumes. Figure 3 shows an example from this dataset. The second dataset is Multiple Sclerosis Human Brain MR Imaging Dataset (MS) (CoBrain analytics, 2018), which is available on the Skoltech CoBrain Analytics platform. This dataset contains 170 manually labeled MRI FLAIR sequences of subjects with multiple sclerosis. All the images were acquired on 1.5T Siemens Magnetom Avanto scanner with slice thickness = 5 mm, slice spacing = 1.5 mm and have resolution 448 × 512 × 22. Figure 4 depicts one sample from this dataset.

### 2.5. Evaluation Methods

Two conventional metrics for semantic segmentation (Clèrigues et al., 2018; Deniz et al., 2018; Kao et al., 2018; Myronenko, 2018) are used to evaluate the model performance—Dice Similarity Coefficient (DSC), also known as *F*_1_-score, and Intersection over union (IoU):

$$\begin{array}{ll} \text{DSC} & =\frac{2TP}{2TP\;+\;FN\;+\;FP}=\frac{2\text{IoU}}{1\;+\;\text{IoU}},\\ \text{IoU} & =\frac{TP}{TP\;+\;FN\;+\;FP}. \end{array}$$

The number of true positive (TR), false negative (FN) and false positive (FP) pixels is calculated for each image and averaged over the whole test set. We compare model predictions with the segmentations, which have been manually created by professional radiologists (Menze et al., 2015) and are considered as ground truth.

### 2.6. Loss

To train U-Net in the non-Bayesian setting, we use a combination of binary cross-entropy and Dice losses. We apply this loss when training all models without Deep Weight Prior: for pre-training on the source dataset, fine-tuning on target dataset and training models with random initialization.

The first component of the loss, binary cross-entropy, is a common loss function for classification problem (Goodfellow et al., 2016). In semantic segmentation setting we classify each pixel of the input image, resulting in the following loss function:

$$\begin{array}{l} L_{CE}=-\underset{i,j}{\sum }y_{i,j}\log{\hat{y}}_{i,j}+\left(1-y_{i,j}\right)\log\left(1-{\hat{y}}_{i,j}\right), \end{array}$$

where ${\hat{y}}_{i,j}$ is a predicted probability for pixel *j* from image *i* to be from the class of interest. Problem with cross-entropy is that it does not account for class imbalance, which usually takes place in semantic segmentation tasks, since background is the most prevalent class. Dice loss, in contrast, is known to be robust to this problem. It is based on Dice Similarity Coefficient and defined as:

$$\begin{array}{l} L_{DICE}=\overset{N}{\underset{i=1}{\sum }}\frac{FN_{i}\;+\;FP_{i}}{2TP_{i}\;+\;FN_{i}\;+\;FP_{i}}=\overset{N}{\underset{i=1}{\sum }}\left(1-DSC^{\left(i\right)}\right). \end{array}$$

The weight of each component in the final combination was chosen experimentally. Since cross-entropy loss resulted in model learning to predict background for all the pixels in most cases, we arrived to the setting where it has a low weight of 0.01:

$$\begin{array}{l} L=0.99L_{DICE}+0.01L_{CE}. \end{array}$$

### 2.7. Experimental Setup

The aim of the experiments is to compare the proposed method (Unet-DWP) with the conventional transfer learning approach: training the model on the small target dataset with pretrained on the source dataset (UNet-PR) or freezing layers in the middle of the network (UNet-PRf) while fine-tuning only the first and the last block of the model to reduce overfitting on a small dataset. As a baseline, we also consider random initialization (UNet-RI), where the model is trained only on the small target dataset. We use initialization introduced in He et al. (2015), also known as He initialization for UNet-RI. The training procedure summarized in the Algorithm 2 for pre-training approaches and in the Algorithm 3 for proposed training with deep prior (UNet-DWP). To compare the proposed methods, we use MS dataset as a source and small subsets of BRATS18 dataset as targets. Both dataset consider the MRI scans of the brain, however with different diseases. The purpose of this setup to show the ability of the method to generalize between diseases. Models performance was compared on the whole tumor segmentation on subsets of BRATS18 volumes, containing 5, 10, 15, or 20 randomly selected images with the fixed test sample size of 50 images. The proposed method is mostly relevant for datasets of small sizes since they do not contain enough samples to train proper network and prior knowledge from a larger dataset should improve the quality.

**Algorithm 2:** Procedure for UNet-PR training on *m* images

**Input:** Dataset to train prior on $D_{prior}:{\left\{\left(x_{i},y_{i}\right)\right\}}_{i=1}^{N}$
**Input:** Target dataset $D_{target}:{\left\{\left(x_{i},y_{i}\right)\right\}}_{i=1}^{n}$
Train one 3D U-Net model on $D_{prior}$ and remember the weights
**for** Iteration ∈ [1, 2, 3] **do**
Split $D_{target}$ on train and test: $D_{target}^{Train}$, $D_{target}^{Test}$
Select *m* images from $D_{target}^{Train}$
Initialize model with the weights trained on $D_{prior}$
Train 3D U-Net on selected images
Evaluate model on $D_{target}^{Test}$

**Algorithm 3:** Procedure of UNet-DWP training on *m* images

**Input:** Dataset to train prior on $D_{prior}:{\left\{\left(x_{i},y_{i}\right)\right\}}_{i=1}^{N}$
**Input:** Target dataset $D_{target}:{\left\{\left(x_{i},y_{i}\right)\right\}}_{i=1}^{n}$
Train 3D U-Net models with different initializations on $D_{prior}$
Collect kernels and split them into seven parts (depending on the input size of the layer)
Train 7 VAE, to use them as implicit prior
**for** Iteration ∈ [1, 2, 3] **do**
Split $D_{target}$ on train and test: $D_{target}^{Train}$, $D_{target}^{Test}$
Select *m* images from $D_{target}^{Train}$
Train 3D U-Net on selected images with DWP
Evaluate model on $D_{target}^{Test}$

#### 2.7.1. U-Net Training Details

All the models on the target dataset were trained on the whole volumes with batch size 2 and without any data augmentation. Table 1 summarize hyperparameters details used during training. For training we use Adam optimizer with initial learning rate 10^−3^. Learning rate is decreased by the factor of 10, when loss on the validation set is not decreasing by more than 10^−4^ during 10 epochs. We stop training the model as soon as learning rate reaches the value 10^−6^. Three different train-test splits of BRATS18 were used for validation in order to verify the robustness of the result. All the experiments were performed on the NVIDIA Tesla V100-SXM2 GPUs.

**Table 1:** U-Net hyperparameters details.

**Parameter**	**Value**
Batch-size	2
Optimizer	Adam
Initial learning rate	10^−3^
LR scheduler	Reduce learning rate when a loss has stopped improving
LR scheduler patience	10
LR scheduler factor	0.1
Max epochs	500
Early stopping criterion	LR == 10^−6^
Test size	50
Train sizes	[5, 10, 15, 20]

**Figure 5:**
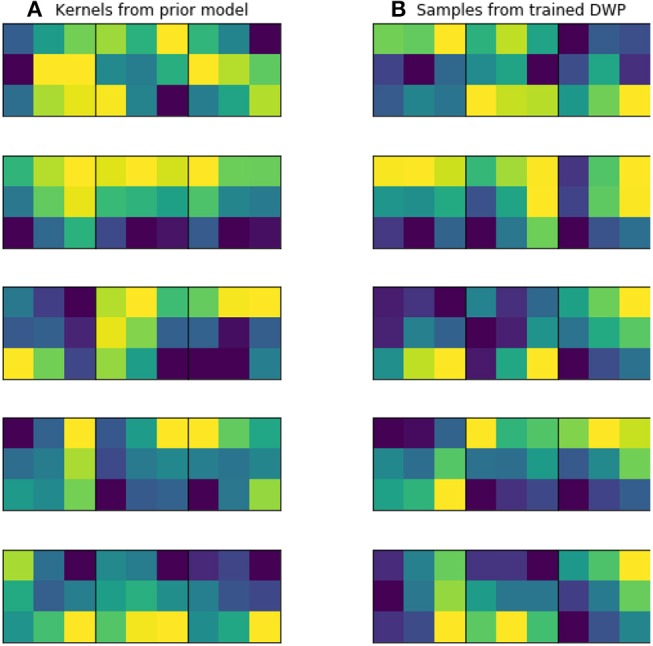
Examples of trained kernels. **(A)** Kernels from U-Net, trained on MS dataset, which were further used to train DWP. **(B)** Samples from trained Deep Weight Prior.

Kernels for further DWP training were collected from U-Net network, trained on the while volumes of the source dataset. Batch size, optimizer and LR scheduler are presented in Table 1. We have applied this setting to train 10 models until convergence, which took on average 100 epochs for one model. To obtain more filters, we have applied cyclical learning rate (Smith, 2017) to obtain 10 more networks. That is we increase learning rate back to 10^−3^ for a converged model and continue training it with the same LR scheduler to converge to a new minimum. As a result, we end up with 20 trained networks with average Dice Score of 0.61 on validation set. As can be seen from the Figure 5, obtained filters have clear structure, which indicates their potential usefulness.

#### 2.7.2. DWP Training Details

To train the DWP prior we should specify the number and architecture of the variational auto-encoders and collect the training set of filters. We train variational autoencoder with latent vector dimention 6. We've used Adam optimizer, batch size of 20 images. All the hyperparameters are presented in the Table 2. Appendix B contains architectures, which were used. We assume that filters from the layers, which take as input images of the same resolution come from the same distribution. As a result, seven Variational Autoencoders were trained and served further as implicit prior distributions for the kernels of the corresponding layers. To obtain the training set of filters U-Net models were trained on the whole MS dataset with random initialization. Afterwards, kernels were collected from trained models to train prior in the form of the Variational Autoencoder.

**Table 2:** DWP hyperparameters details.

**Parameter**	**Value**
Batch-size	20
Optimizer	Adam
Initial learning rate	10^−3^
LR scheduler	Reduce learning rate when a loss has stopped improving
LR scheduler patience	15
LR scheduler factor	0.1
Max epochs	500
Early stopping criterion	LR == 10^−6^
Latent dimension	6

## 3. Results

**Figure 6:**
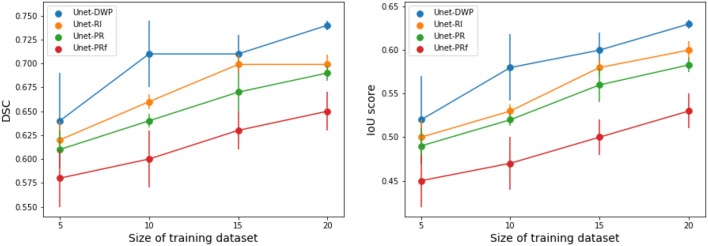
Segmentation accuracy on BRATS18 dataset for various train sample size, calculated for three different splits.

Each model (UNet-RI, UNet-DWP, UNet-PR and UNet-PRf) was estimated at three different random train/test splits. For a fixed test sample of 50 images 5, 10, 15, and 20 images were selected for training, and on each sample, three models were estimated. Tables 3, 4 and Figure 6 summarize the obtained results. UNet-RI stands for the model trained with the random initialization, UNet-PR and UNet-PRf are transfer learning approaches (in the second case, weights of the middle layers were frozen), where U-Net was pre-trained on MS dataset and, finally, UNet-DWP is a model trained with Deep Weight Prior. We calculate mean DSC and IoU metrics for different train-test splits and its standard deviation, which is given in the brackets.

**Table 3:** Mean Dice Similarity Score for the different subsets of BRATS18 dataset.

**Train size**	**UNet-DWP (ours)**	**UNet-PR**	**UNet-PRf**	**UNet-RI**
5	**0.64** (0.05)	0.61 (0.02)	0.58 (0.03)	0.62 (0.02)
10	**0.71** (0.04)	0.64 (0.01)	0.60 (0.03)	0.66 (0.01)
15	**0.71** (0.02)	0.67 (0.02)	0.63 (0.02)	0.70 (0.02)
20	**0.74** (0.01)	0.69 (0.01)	0.65 (0.02)	0.70 (0.01)

*Best results are in bold*.

**Table 4:** Mean Intersection over Union for the different subsets of BRATS18 dataset.

**Train size**	**UNet-DWP (ours)**	**UNet-PR**	**UNet-PRf**	**UNet-RI**
5	**0.52** (0.05)	0.49 (0.02)	0.45 (0.03)	0.50 (0.02)
10	**0.58** (0.05)	0.52 (0.01)	0.47 (0.03)	0.53 (0.01)
15	**0.60** (0.02)	0.56 (0.02)	0.50 (0.02)	0.58 (0.02)
20	**0.63**(0.01)	0.58 (0.01)	0.53 (0.02)	0.60 (0.01)

*Best results are in bold*.

We can see that models trained with DWP noticeably outperformed both randomly initialized and pre-trained U-Net for all the training sizes. We observe higher variability in prediction accuracy for the problems with smaller sample sizes, which shrinks as training dataset grows, and the superiority of UNet-WDP becomes clearer. It is also worth mentioning that the pre-trained mode where part of the weights were frozen fails. We believe that this means that information from other diseases is not relevant for the new task by default, and without fine-tuning of the whole network, we are not able to achieve consistent results.

**Figure 7:**
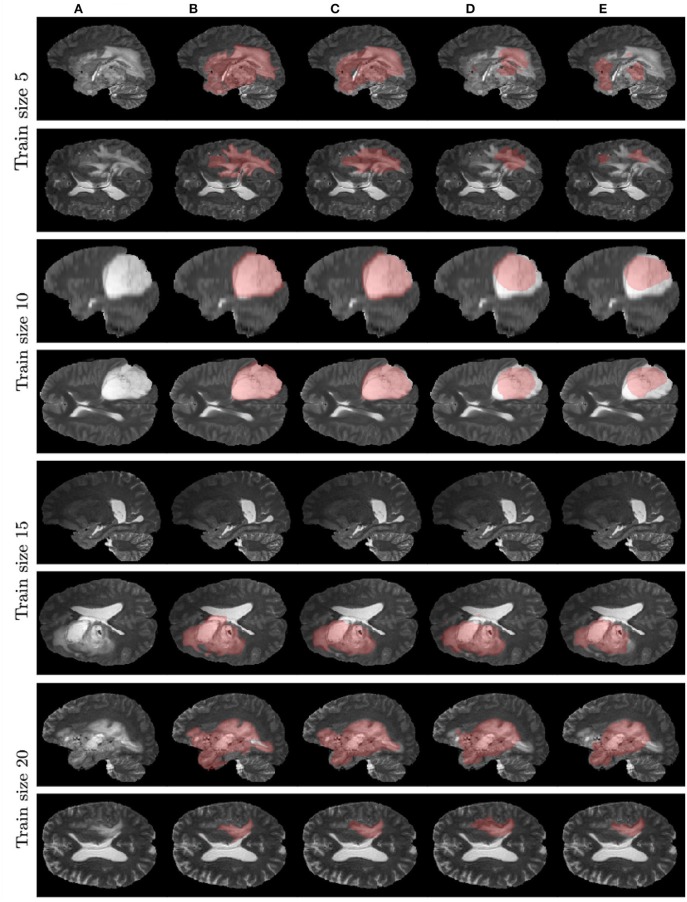
Examples of models' predictions on test samples, compared to ground truth segmentation. **(A)** Test MRI. **(B)** Ground truth segmentation. **(C)** UNet-DWP. **(D)** UNet-PR. **(E)** UNet-RI.

Figure 7 contains example predictions of different models (Figures 7C–E) along with ground truth segmentations (Figure 7B). Each row corresponds to different training sample size. For example, for the model trained on 10 images, there is a notable difference in tumor coverage for UNet-DWP and UNet-PR models, which results in DSC of 0.92 for the first model and 0.74 for the second. On other images we may also note, that model with DWP manages to cover more relevant areas.

It is worth mentioning, that transfer learning model on average performs even worse than the model without any prior knowledge about the data. This result is quite surprising, but it can be explained by strong disease specificity of the data. Even from the examples in Figures 3, 4 it can be seen, that datasets differ not only in the shapes of the target segmentation (plaques of multiple sclerosis are much smaller and difficult to notice that brain tumor) but also in resolution, contrast and preprocessing method, as a result, after corresponding initialization, fine-tuning may converge to a worse solution.

Figure 5 illustrates prior over the weights, that was used for the given experiment. Figure 5A contains kernels of the U-Net, trained on the MS dataset. Since the dataset is big enough, they are not noisy and have clear structure, as it was expected. Figure 5B depicts samples from Variational Autoencoder, which was later used as an implicit prior distribution. Even though samples from the Deep Weight Prior on the right are not identical to the real kernels on the left, they still have similar structure and we can assume that the VAE managed to grasp a proper distribution.

## 4. Discussion

The proposed method can be used for knowledge transfer between medical imaging data from different domains, resulting in performance improvement over conventional transfer learning. This method is based on the deep Bayesian approach and exploits implicit prior distribution over convolutional filters.

Our approach is not limited to a specific task and can be applied to such problems as classification, detections or any other, where convolutional neural networks are used. But we believe that it is the most relevant for the semantic segmentation problem. There are plenty of challenges in this area. One of the most significant is that manual segmentation of MRI volumes, which is needed to train any supervised model, is very expensive. The reason is that it requires the work of several professional radiologists and each image should be labeled several times by different people to reduce ambiguity. For instance, it takes around 60 min for a radiologist to label one scan of the patient with a brain tumor, resulting in 4 h of work per observation (Menze et al., 2015). Moreover, institutions are often hesitant to share data with external collaborators because of patient privacy, as well as ethical and legal considerations. As a result, there are very few publicly available datasets, and they are often tiny, up to 5 or 10 images. Besides, data is highly disease-specific, making conventional transfer learning technique inefficient for cases, when source dataset, used for initial model training, has a different domain (another illness, MRI modality and preprocessing method), which is confirmed in our experiments.

The most popular model for semantic segmentation is volumetric U-Net (Deniz et al., 2018; Guerrero et al., 2018; Livne et al., 2019). The idea behind this architecture is quite simple; it is based on conventional U-Net model for semantic segmentation. The main advantage of this models is that it proceeds the whole 3D image, using 3D convolutions, instead of working with 2-dimensional slices separately. It is also quite common to use different heuristic regularization techniques: VAE regularization, (Myronenko, 2018), anatomically constrained U-Net (Degel et al., 2018; Oktay et al., 2018).

In this work, we compare three methods for semantic segmentation of a brain tumor on small datasets of size from 5 to 20. In the first approach, we train 3D U-Net from scratch, using the information only from a given small dataset, in the second approach we firstly train a model on a large dataset with multiple sclerosis segmentation and then use trained kernels to initialize model for brain tumor segmentation. Finally, we propose a new approach to transfer information using Deep Weight Prior—implicit prior distribution, also learned on a large dataset with multiple sclerosis and applied to train tumor segmentation network. We have shown that the proposed approach outperforms both simple and fine-tuned models. Presumably, transfer learning approach fails in this case because prior was trained on the samples with different illness and information about it is not a proper initialization for a new task. At the same time, Deep Weight Prior ensures that kernels come from similar distribution, bringing up structure into convolutional filters. Even though all the experiments were performed on a simple U-Net model, it can be applied to any other architecture with a more complicated structure.

### 4.1. Extra Experiments

During our experiments, we aimed at using datasets containing the same organs to make sure that the source data has enough relevant information to transfer to the target one. But of course, the proposed method is not limited to the specific part of the human body and can be applied to other organs as well. To test this hypothesis, we performed additional experiments on the dataset, containing CT scans of the liver from the medical decathlon segmentation challenge (Simpson et al., 2019). As a source Task08_HepaticVessel dataset was used, containing 443 CT scans of patients with liver tumors. As a target dataset subsets of Task03_Liver and Task09_Spleen datasets were used. The first target dataset is closer to the source one since it contains 201 CT images of patients with a liver tumor. In the second dataset the aim is to segment spleen on the CT scans for 41 patients, which makes transfer learning from the source dataset a more challenging task.

The purpose of this additional experiment was to provide evidence that the proposed method can be successfully applied not only to the brain but also to other organs. We did not tune the architecture to reach state-of-the-art performance for the specific dataset. Instead, we applied the same architecture, experimental setups as in the main part of the paper. The only difference was that due to the large image size, we had to use patches instead of the whole volumes ([40, 400, 400] for the source, [192, 192, 192] for the first target dataset and [24, 480, 480] for the second target dataset).

Preliminary results that we have obtained are quite promising. They are presented in Table 5 and show that for both target datasets U-Net with Deep Weight Prior performs better than the competitors in most cases. We believe that this part could be further improved, by tuning the architecture and adding more datasets for comparison.

**Table 5:** Mean Dice Similarity Score for the subsets of Task03_Liver and Task09_Spleen datasets.

	**Task03_Liver**			**Task09_Spleen**		
**Train size**	**UNet-DWP (ours)**	**UNet-RI**	**UNet-PR**	**UNet-DWP (ours)**	**UNet-RI**	**UNet-PR**
5	0.275	**0.284**	0.209	**0.467**	0.391	0.105
10	**0.328**	0.293	0.052	**0.625**	0.584	0.239
15	**0.389**	0.306	0.243	0.556	**0.579**	0.302
20	**0.353**	0.336	0.156	**0.649**	0.566	0.459

*Best results are in bold*.

The reasonable question arises is the necessity for transferring when a relatively large dataset is available. Hence, we consider the additional experiment of transfer learning from the MS dataset as the source to the BRATS18 as the target, while 100 of samples are available from the target dataset.

Taking into account standard deviation, results, presented in Table 6 are quite close to each other. As it was expected, our method converges to the UNet-RI result as a number of training samples increases, since in this case knowledge transfer becomes less useful because there is enough information in the target dataset to train a proper network (Lu, 2017).

**Table 6:** Mean Dice Similarity Score for the experiments with large available target dataset (MS-BRATS18).

**Train size**	**UNet-DWP (ours)**	**UNet-PR**	**UNet-PRf**	**UNet-RI**
100	0.76 (0.01)	0.79 (0.01)	0.77 (0.01)	0.77 (0.01)

Further research on the topic may include experiments with knowledge transfer from other problem settings, e.g., from classification to segmentation and vice versa. The first setting is of higher interest, since there are usually more observations in classification datasets and there are more of them available for different diseases, making it a more accessible source of prior knowledge.

## Author Contributions

AK, EE, and EB contributed to conception and design of the study. AK performed the statistical analysis. AK and EE wrote the first draft of the manuscript. All authors contributed to manuscript revision, read and approved the submitted version.

### Conflict of Interest Statement

The authors declare that the research was conducted in the absence of any commercial or financial relationships that could be construed as a potential conflict of interest.

## References

[B1] Atanov A. Ashukha A. Struminsky K. Vetrov D. Welling M. (2018). The deep weight prior. arXiv:1810.06943.

[B2] Bakas S. Akbari H. Sotiras A. Bilello M. Rozycki M. Kirby J. S.. (2017). Advancing the cancer genome atlas glioma mri collections with expert segmentation labels and radiomic features. Sci. Data 4:170117. 10.1038/sdata.2017.117 28872634 PMC5685212

[B3] Christodoulidis S. Anthimopoulos M. Ebner L. Christe A. Mougiakakou S. (2017). Multisource transfer learning with convolutional neural networks for lung pattern analysis. IEEE J. Biomed. Health Informat. 21, 76–84. 10.1109/JBHI.2016.2636929 28114048

[B4] Çiçek Ö Abdulkadir A Lienkamp S. S Brox T Ronneberger O. (2016). 3D U-Net: learning dense volumetric segmentation from sparse annotation, in Medical Image Computing and Computer-Assisted Intervention – MICCAI 2016. MICCAI 2016. Lecture Notes in Computer Science, Vol. 9901, eds Ourselin S. Joskowicz L. Sabuncu M. Unal G. Wells W. (Cham: Springer). 10.1007/978-3-319-46723-8_49

[B5] Clèrigues A. Valverde S. Bernal J. Freixenet J. Oliver A. Lladó X. (2018). SUNet: a deep learning architecture for acute stroke lesion segmentation and outcome prediction in multimodal mri. arXiv:1810.13304.

[B6] CoBrain analytics (2018). Multiple Sclerosis Human Brain MR Imaging Dataset. Available online at: https://app.cobrain.io/datasets/2c683256-6dcd-47bc-9399-34e166c3fc71

[B7] Davatzikos C. Resnick S. M. Wu X. Parmpi P. Clark C. M. (2008). Individual patient diagnosis of AD and FTD via high-dimensional pattern classification of MRI. Neuroimage 41, 1220–1227. 10.1016/j.neuroimage.2008.03.050 18474436 PMC2528893

[B8] Davuluri P. Wu J. Tang Y. Cockrell C. H. Ward K. R. Najarian K.. (2012). Hemorrhage detection and segmentation in traumatic pelvic injuries. Comput. Math. Methods Med. 2012:898430. 10.1155/2012/898430 22919433 PMC3418697

[B9] Degel M. A. Navab N. Albarqouni S. (2018). Domain and geometry agnostic CNNs for left atrium segmentation in 3d ultrasound. arXiv:1805.00357. 10.1007/978-3-030-00937-3_72

[B10] Deniz C. M. Xiang S. Hallyburton R. S. Welbeck A. Babb J. S. Honig S.. (2018). Segmentation of the proximal femur from MR images using deep convolutional neural networks. Sci. Rep. 8:16485. 10.1038/s41598-018-34817-6 30405145 PMC6220200

[B11] Elsayed G. F. Goodfellow I. Sohl-Dickstein J. (2018). Adversarial reprogramming of neural networks. arXiv:1806.11146.

[B12] Ghafoorian M. Mehrtash A. Kapur T. Karssemeijer N. Marchiori E. Pesteie M. (2017). Transfer learning for domain adaptation in MRI: application in brain lesion segmentation, in Medical Image Computing and Computer Assisted Intervention - MICCAI 2017. Lecture Notes in Computer Science, Vol. 10435, eds Descoteaux M. Maier-Hein L. Franz A. Jannin P. Collins D. Duchesne S. (Cham: Springer). 10.1007/978-3-319-66179-7_59

[B13] Gong T Liu R. Tan C. L. Farzad N. Lee C. K. Pang B. C. (2007). Classification of CT brain images of head trauma, in Pattern Recognition in Bioinformatics. PRIB 2007. Lecture Notes in Computer Science, Vol. 4774, eds Rajapakse J. C. Schmidt B. Volkert G. (Berlin; Heidelberg: Springer). 10.1007/978-3-540-75286-8_38

[B14] Goodfellow I. Bengio Y. Courville A. (2016). Deep Learning. Cambridge: MIT Press.

[B15] Guerrero R. Qin C. Oktay O. Bowles C. Chen L. Joules R.. (2018). White matter hyperintensity and stroke lesion segmentation and differentiation using convolutional neural networks. NeuroImage 17, 918–934. 10.1016/j.nicl.2017.12.022 29527496 PMC5842732

[B16] Hammers A. Heckemann R. Koepp M. J. Duncan J. S. Hajnal J. V. Rueckert D.. (2007). Automatic detection and quantification of hippocampal atrophy on mri in temporal lobe epilepsy: a proof-of-principle study. Neuroimage 36, 38–47. 10.1016/j.neuroimage.2007.02.031 17428687

[B17] Han Y. Yoo J. Kim H. H. Shin H. J. Sung K. Ye J. C. (2018). Deep learning with domain adaptation for accelerated projection-reconstruction MR. Magn. Reson. Med. 80, 1189–1205. 10.1002/mrm.27106 29399869

[B18] Havaei M Guizard N Larochelle H Jodoin P. M (2016). Deep learning trends for focal brain pathology segmentation in MRI, in Machine Learning for Health Informatics. Lecture Notes in Computer Science, Vol. 9605, ed Holzinger A. (Cham: Springer). 10.1007/978-3-319-50478-0_6

[B19] He K. Zhang X. Ren S. Sun J. (2015). Delving deep into rectifiers: surpassing human-level performance on imagenet classification, in Proceedings of the IEEE International Conference on Computer Vision (Santiago), 1026–1034.

[B20] Hoffman M. D. Blei D. M. Wang C. Paisley J. (2013). Stochastic variational inference. J. Mach. Learn. Res. 14, 1303–1347.

[B21] Isensee F. Petersen J. Klein A. Zimmerer D. Jaeger P. F. Kohl S. (2018). nnu-net: self-adapting framework for u-net-based medical image segmentation. arXiv:1809.10486. 10.1007/978-3-658-25326-4_7

[B22] Ivanov S. Sharaev M. Artemov A. Kondratyeva E. Sushchinskaya S. Burnaev E. (2018). Learning connectivity patterns via graph kernels for fmri-based depression diagnostics, in Proceedings of IEEE International Conference on Data Mining Workshops (ICDMW) (Singapore), 308–314.

[B23] Jordan M. I. Ghahramani Z. Jaakkola T. S. Saul L. K. (1999). An introduction to variational methods for graphical models. Mach. Learn. 37, 183–233. 10.1023/A:1007665907178

[B24] Kao P.-Y. Ngo T. Zhang A. Chen J. W. Manjunath B. (2018). Brain tumor segmentation and tractographic feature extraction from structural mr images for overall survival prediction. arXiv:1807.07716. 10.1007/978-3-030-11726-9_12

[B25] Kingma D. P. Salimans T. Welling M. (2015). Variational dropout and the local reparameterization trick, in 29th Annual Conference on Neural Information Processing Systems 2015, Vol. 3, eds Cortes C. Lawrence N. D. Lee D. D. Sugiyama M. Garnett R. (Montreal, QC), 2575–2583.

[B26] Kingma D. P. Welling M. (2014). Auto-encoding variational Bayes, in Conference Proceedings: Papers Accepted to the International Conference on Learning Representations, (ICLR) (Ithaca, NY: ICLR).

[B27] Kohl S. Bonekamp D. Schlemmer H.-P. Yaqubi K. Hohenfellner M. Hadaschik B. (2017). Adversarial networks for the detection of aggressive prostate cancer. arXiv:1702.08014.

[B28] Li H. Parikh N. A. He L. (2018). A novel transfer learning approach to enhance deep neural network classification of brain functional connectomes. Front. Neurosci. 12:491. 10.3389/fnins.2018.00491 30087587 PMC6066582

[B29] Litjens G. Kooi T. Bejnordi B. E. Setio A. A. A. Ciompi F. Ghafoorian M.. (2017). A survey on deep learning in medical image analysis. Med. Image Anal. 42, 60–88. 10.1016/j.media.2017.07.005 28778026

[B30] Livne M. Rieger J. Aydin O. U. Taha A. A. Akay E. M. Kossen T.. (2019). A U-net deep learning framework for high performance vessel segmentation in patients with cerebrovascular disease. Front. Neurosci. 13:97. 10.3389/fnins.2019.00097 30872986 PMC6403177

[B31] Lu Y. (2017). On the bernstein-von mises theorem for high dimensional nonlinear bayesian inverse problems. arXiv:1706.00289.

[B32] Margeta J. Criminisi A. Cabrera Lozoya R. Lee D. C. Ayache N. (2017). Fine-tuned convolutional neural nets for cardiac MRI acquisition plane recognition. Comput. Methods Biomech. Biomed. Eng. 5, 339–349. 10.1080/21681163.2015.1061448

[B33] Menze B. H. Jakab A. Bauer S. Kalpathy-Cramer J. Farahani K. Kirby J.. (2015). The multimodal brain tumor image segmentation benchmark (brats). IEEE Trans. Med. Imaging 34:1993. 10.1109/TMI.2014.2377694 25494501 PMC4833122

[B34] Milletari F. Navab N. Ahmadi S.-A. (2016). V-net: fully convolutional neural networks for volumetric medical image segmentation, in 3D Vision (3DV), 2016 Fourth International Conference on (Stanford, CA: IEEE), 565–571. 10.1109/3DV.2016.79

[B35] Mlynarski P. Delingette H. Criminisi A. Ayache N. (2018). Deep learning with mixed supervision for brain tumor segmentation. arXiv:1812.04571. 10.1117/1.JMI.6.3.034002 PMC6689144 31423456

[B36] Myronenko A. (2018). 3d MRI brain tumor segmentation using autoencoder regularization. arXiv:1810.11654. 10.1007/978-3-030-11726-9_28

[B37] Neklyudov K. Molchanov D. Ashukha A. Vetrov D. P. (2017). Structured bayesian pruning via log-normal multiplicative noise, in Advances in Neural Information Processing Systems (Vancouver, BC), 6775–6784.

[B38] Oktay O. Ferrante E. Kamnitsas K. Heinrich M. Bai W. Caballero J.. (2018). Anatomically constrained neural networks (ACNNs): application to cardiac image enhancement and segmentation. IEEE Trans. Med. Imaging 37, 384–395. 10.1109/TMI.2017.2743464 28961105

[B39] Pan S. J. Yang Q. (2010). A survey on transfer learning. IEEE Trans. Knowl. Data Eng. 22, 1345–1359. 10.1109/TKDE.2009.191

[B40] Pominova M. Artemov A. Sharaev M. Kondrateva E. Cichocki A. Burnaev E. (2018). Voxelwise 3d convolutional and recurrent neural networks for epilepsy and depression diagnostics from structural and functional mri data, in Proceedings of the IEEE International Conference on Data Mining Workshops (ICDMW) (Singapore), 299–307.

[B41] Rey D. Subsol G. Delingette H. Ayache N. (2002). Automatic detection and segmentation of evolving processes in 3d medical images: application to multiple sclerosis. Med. Image Anal. 6, 163–179. 10.1016/S1361-8415(02)00056-7 12045002

[B42] Ronneberger O Fischer P Brox T (2015). U-Net: convolutional networks for biomedical image segmentation, in Medical Image Computing and Computer-Assisted Intervention – MICCAI 2015. MICCAI 2015. Lecture Notes in Computer Science, Vol. 9351, eds Navab N. Hornegger J. Wells W. Frangi A. (Cham: Springer). 10.1007/978-3-319-24574-4_28

[B43] Shah M. P Merchant S. N Awate S. P (2018). MS-Net: mixed-supervision fully-convolutional networks for full-resolution segmentation, in Medical Image Computing and Computer Assisted Intervention – MICCAI 2018. MICCAI 2018. Lecture Notes in Computer Science, Vol. 11073, eds Frangi A. Schnabel J. Davatzikos C. Alberola-López C. Fichtinger G. (Cham: Springer). 10.1007/978-3-030-00937-3_44

[B44] Sharaev M. Andreev A. Artemov A. Burnaev E. Kondratyeva E. Sushchinskaya S. (2018a). Pattern recognition pipeline for neuroimaging data, in Artificial Neural Networks in Pattern Recognition, eds Pancioni L. Schwenker F. Trentin E. (Cham: Springer International Publishing), 306–319.

[B45] Sharaev M. Artemov A. Kondratyeva E. Sushchinskaya S. Burnaev E. Bernstein A. (2018b). Mri-based diagnostics of depression concomitant with epilepsy: in search of the potential biomarkers, in Proceedings of IEEE 5th International Conference on Data Science and Advanced Analytics (Turin), 555–564.

[B46] Sheline Y. I. (2000). 3d MRI studies of neuroanatomic changes in unipolar major depression: the role of stress and medical comorbidity. Biol. Psychiatry 48, 791–800. 10.1016/S0006-3223(00)00994-X 11063975

[B47] Simpson A. L. Antonelli M. Bakas S. Bilello M. Farahani K. van Ginneken B. (2019). A large annotated medical image dataset for the development and evaluation of segmentation algorithms. arXiv [Preprint]. arXiv:1902.09063.

[B48] Smith L. N. (2017). Cyclical learning rates for training neural networks, in 2017 IEEE Winter Conference on Applications of Computer Vision (WACV) (Santa Rosa, CA: IEEE), 464–472.

[B49] Van Opbroek A. Ikram M. A. Vernooij M. W. De Bruijne M. (2015). Transfer learning improves supervised image segmentation across imaging protocols. IEEE Trans. Med. Imaging 34, 1018–1030. 10.1109/TMI.2014.2366792 25376036

[B50] Wachinger C. Reuter M. Klein T. (2018). DeepNAT: deep convolutional neural network for segmenting neuroanatomy. NeuroImage 170, 434–445. 10.1016/j.neuroimage.2017.02.035 28223187 PMC5563492

[B51] Wilson G. Cook D. J. (2018). Adversarial transfer learning. arXiv:1812.02849.

[B52] Zhou Z. Shin J. Zhang L. Gurudu S. Gotway M. Liang J. (2017). Fine-tuning convolutional neural networks for biomedical image analysis: actively and incrementally, in Proceedings of the IEEE Conference on Computer Vision and Pattern Recognition (Santa Rosa, CA), 7340–7351. 10.1109/CVPR.2017.506 PMC6191179 30337799

