# Supplementary material for: Bayesian Generative Models for Knowledge Transfer in MRI Semantic Segmentation Problems
Source: Front Neurosci. 2019 Aug 21;13:844. doi: 10.3389/fnins.2019.00844 (PMC6712162; doi:10.3389/fnins.2019.00844)
Supplement: Supplementary file 1 [file Data_Sheet_1.pdf]

Bayesian Generative Models for Knowledge Transfer in MRI Semantic Segmentation Problems: Appendix

Anna Kuzina *
a.kuzina@skoltech.ru

Evgenii Egorov *
e.egorov@skoltech.ru

Evgeny Burnaev *
e.burnaev@skoltech.ru

A. Stohastic Variational Inference

Variational inference (Jordan et al., 1999) introduces approximate posterior distribution $q_\theta(w)$ from some parametric family, e.g. fully factorized Gaussian, and solve optimization problem, minimizing Kullback–Leibler divergence between true posterior distribution $p(w|\mathcal{D})$ and variational approximation $q_\theta(w)$ with respect to parameters θ.

$$\min \mathrm{KL}\left(q_\theta(w))||p(w|\mathcal{D})\right). \tag{1}$$

Where Kullback–Leibler divergence, or KL-divergence is defined as:

$$\mathrm{KL}\left(q(x))||p(x)\right) = -\int q(x) \log \left(\frac{p(x)}{q(x)}\right).$$

Note that equation (1) still contains posterior distribution, which is not known. Let us rewrite this equation in the following way:

$$\begin{aligned}\mathrm{KL}\left(q_\theta(w))||p(w|\mathcal{D})\right) &= \mathbb{E}_{q_\theta(w)} \log \frac{q_\theta(w)p(\mathcal{D})}{p(\mathcal{D}|w)p(w)} = \\ &= \log p(\mathcal{D}) + \mathbb{E}_{q_\theta(w)} \log \frac{q_\theta(w)}{p(w)} - \mathbb{E}_{q_\theta(w)} \log p(\mathcal{D}|w) = \\ &= \log p(\mathcal{D}) - \mathcal{L}(\theta).\end{aligned}$$

Above we have received a decomposition of the marginal log-likelihood into two components: the first one is KL-divergence between exact posterior and its variational approximation, while the second one is so-called evidence lower bound (ELBO, $\mathcal{L}(\theta)$).

$$\max_\theta \log p(\mathcal{D}) = \max_\theta \left[\mathrm{KL}\left(q_\theta(w))||p(w|\mathcal{D})\right) + \mathcal{L}(\theta)\right].$$

If variational posterior is precisely equal to the true posterior, KL-divergence is zero and ELBO coincides with marginal log-likelihood. Since KL-divergence is always non-negative, ELBO cannot be greater than $\log p(\mathcal{D})$ and thus problem reduces to ELBO maximization.

*Skolkov Institute of Science and Technology, Center for Computational and Data-Intensive Science and Engineering

$$\mathcal{L}(\theta) = \mathbb{E}_{q_\theta(w)} \log p(\mathcal{D}|w) - \mathbb{E}_{q_\theta(w)} \log \frac{q_\theta(w)}{p(w)} = \mathcal{L}_\mathcal{D} - \mathrm{KL}\left(q_\theta(w)||p(w)\right).$$

The first part of the target function is data term $\mathcal{L}_\mathcal{D}$ also referred to as a reconstruction error. It is in charge of prediction quality, forcing the model to fit the data. Second term — Kullback–Leibler divergence between a variational distribution and prior over parameters of the model requires posterior distribution to be as close as possible to the prior, serving among other things as a regularization.

In complex models, such as neural networks, it is not trivial to compute gradients of the data term $\mathcal{L}_\mathcal{D}$. In practice one may overcome this difficulty with the help of sampling and reparametrization trick, resulting in so-called doubly stochastic variational inference (Kingma et al., 2015). Let $\mathcal{D}^*$ be minibatch of size $M < N$ and $w = f(\theta, \varepsilon_i)$ a representation of the parametric random variable $w \sim q_\theta(w)$ as a deterministic function of the non-parametric noise $\varepsilon \sim p(\varepsilon)$. Then the unbiased Monte Carlo estimate of the data term $\mathcal{L}_\mathcal{D}$ has the following form:

$$\mathcal{L}_\mathcal{D} \approx \mathcal{L}_{\mathcal{D}^*} = \frac{N}{M} \sum_{i=1}^{M} \log p(\mathcal{D}_i | f(\theta, \widehat{\varepsilon}_i)), \quad \widehat{\varepsilon}_i \sim p(\varepsilon).$$

We apply doubly stochastic variational inference framework(Kingma et al., 2015) to the U-net model. Dataset in this case contains pairs of images $\{x_i\}_{i=1}^N$ and their masks $\{y_i\}_{i=1}^N$. All the parameters of the model are of the form of convolutional filters $(w^{(1)}, \ldots, w^{(L)})$, where L is the number of convolutional layers. We assume that both variational approximation $q_\theta(w)$ and prior distribution $p(w)$ are factorized over layers, input and output channels:

$$q_\theta(w) = \prod_{i=1}^{L} \prod_{p=1}^{C_{inp}^{(i)}} \prod_{k=1}^{C_{out}^{(i)}} q_{\theta_{ipk}}(w_{p,k}^{(i)}),$$
$$p(w) = \prod_{i=1}^{L} \prod_{p=1}^{C_{inp}^{(i)}} \prod_{k=1}^{C_{out}^{(i)}} p(w_{p,k}^{(i)}).$$

where $C_{inp}^{(i)}, C_{out}^{(i)}$ — the number of input and output channels on the i-th layer of the network.

Taking into account both reparametrization trick and factorization of the distributions, the final optimization task is the follwing:

$$\max_\theta \mathcal{L}(\theta) \approx \max_\theta \mathcal{L}_{\mathcal{D}^*} - \sum_{i,p,k} \mathrm{KL}\left(q_{\theta_{ipk}}(w_{p,k}^{(i)}||p(w_{p,k}^{(i)})\right).$$

B. Architecture details

3D U-Net

```
ConvBlock(in_channels, out_channels, s) =
    Sequential(
        (0): InstanceNorm3d(in_channels)
        (1): ReLU()
        (2): Conv3d(in_channels, out_channels, kernel_size=(3, 3, 3), stride=(s, s, s)))

  UNet3D(
  (init_conv): Conv3d(1, 16, kernel_size=(3, 3, 3), stride=(1, 1, 1))
  (down1): BasicDownBlock(
    (conv_1): ConvBlock(16, 32, 2)
    (conv_2): ConvBlock(32, 32, 1)
    (down): ConvBlock(16, 32, 2)
    )
  (down2): BasicDownBlock(
    (conv_1): ConvBlock(32, 32, 1)
    (conv_2): ConvBlock(32, 32, 1)
  )
  (down3): BasicDownBlock(
    (conv_1): ConvBlock(32, 32, 2)
    (conv_2): ConvBlock(32, 32, 1)
    (down): ConvBlock(32, 32, 2)
  )
  (down4): BasicDownBlock(
    (conv_1): ConvBlock(32, 32, 1)
    (conv_2): ConvBlock(32, 32, 1)
  )
  (down5): BasicDownBlock(
    (conv_1): ConvBlock(32, 64, 2)
    (conv_2): ConvBlock(64, 64, 1)
    (down): ConvBlock(32, 64, 2)
  (down6): BasicDownBlock(
    (conv_1): ConvBlock(64, 64, 1)
    (conv_2): ConvBlock(64, 64, 1)
  )
  (up1): BasicUpBlock(
    (upsample): Sequential(
      (0): ConvBlock(64, 32, 1)
      (1): Upsample(scale_factor=2.0, mode=trilinear)
    )
    (conv_1): ConvBlock(32, 32, 1)
    (conv_2): ConvBlock(32, 32, 1)
  )
  (up2): BasicUpBlock(
    (upsample): Sequential(
      (0): ConvBlock(32, 32, 1)
      (1): Upsample(scale_factor=2.0, mode=trilinear)
    )
    (conv_1): ConvBlock(32, 32, 1)
```

```
    (conv_2): ConvBlock(32, 32, 1)
  )
  (up3): BasicUpBlock(
    (upsample): Sequential(
      (0): ConvBlock(32, 16, 1)
      (1): Upsample(scale_factor=2.0, mode=trilinear)
    )
    (conv_1): ConvBlock(16, 16, 1)
    (conv_2): ConvBlock(16, 16, 1)
  )
  (out): Conv3d(16, 2, kernel_size=(1, 1, 1), stride=(1, 1, 1))
)
```

VAE for DWP

```
Kernel_3D_VAE(
  (encode): Sequential(
    (0): Conv3d(1, 32, kernel_size=(3, 3, 3), stride=(1, 1, 1))
    (1): MaxPool3d(kernel_size=2)
    (2): ELU(alpha=1.0)
    (3): Conv3d(32, 64, kernel_size=(3, 3, 3), stride=(1, 1, 1))
    (4): MaxPool3d(kernel_size=2)
    (5): ELU(alpha=1.0)
    (6): Conv3d(64, 128, kernel_size=(1, 1, 1), stride=(1, 1, 1))
    (7): ELU(alpha=1.0)
    (8): Flatten()
  )
  (latent_mu): Linear(in_features=128, out_features=6)
  (latent_logsigma): Linear(in_features=128, out_features=6)
  (linear): Linear(in_features=6, out_features=128)
  (decode): Sequential(
    (0): Conv3d(128, 128, kernel_size=(3, 3, 3), stride=(1, 1, 1))
    (1): ELU(alpha=1.0)
    (2): ConvTranspose3d(128, 128, kernel_size=(3, 3, 3), stride=(1, 1, 1))
    (3): ELU(alpha=1.0)
    (4): ConvTranspose3d(128, 64, kernel_size=(1, 1, 1), stride=(1, 1, 1))
    (5): ELU(alpha=1.0)
    (6): ConvTranspose3d(64, 32, kernel_size=(1, 1, 1), stride=(1, 1, 1))
    (7): ELU(alpha=1.0)
  )
  (reconstruction_mu): Sequential(
    (0): ConvTranspose3d(32, 1, kernel_size=(1, 1, 1), stride=(1, 1, 1))
    (1): Tanh()
  )
  (reconstruction_logsigma): Sequential(
    (0): ConvTranspose3d(32, 1, kernel_size=(1, 1, 1), stride=(1, 1, 1))
    (1): Tanh()
  ))
```

References

Michael I Jordan, Zoubin Ghahramani, Tommi S Jaakkola, and Lawrence K Saul. An introduction to variational methods for graphical models. *Machine learning*, 37(2):183–233, 1999.

Durk P Kingma, Tim Salimans, and Max Welling. Variational Dropout and the Local Reparameterization Trick. *Advances in Neural Information Processing Systems*, pages 2575–2583, 2015.
